# Comparison Analysis of Coal Biodesulfurization and Coal's Pyrite Bioleaching with *Acidithiobacillus ferrooxidans*

**DOI:** 10.1155/2013/184964

**Published:** 2013-10-27

**Authors:** Fen-Fen Hong, Huan He, Jin-Yan Liu, Xiu-Xiang Tao, Lei Zheng, Yi-Dong Zhao

**Affiliations:** ^1^Key Laboratory of Coal Processing and Efficient Utilization of Ministry of Education, School of Chemical Engineering and Technology, China University of Mining and Technology, Xuzhou 221116, China; ^2^College of Zijin Mining, Fuzhou University, Fuzhou 350108, China; ^3^Beijing Synchrotron Radiation Facility, Institute of High Energy Physics, Chinese Academy of Science, Beijing 100049, China

## Abstract

*Acidithiobacillus ferrooxidans* (*A. ferrooxidans*) was applied in coal biodesulfurization and coal's pyrite bioleaching. The result showed that *A. ferrooxidans* had significantly promoted the biodesulfurization of coal and bioleaching of coal's pyrite. After 16 days of processing, the total sulfur removal rate of coal was 50.6%, and among them the removal of pyritic sulfur was up to 69.9%. On the contrary, after 12 days of processing, the coal's pyrite bioleaching rate was 72.0%. SEM micrographs showed that the major pyrite forms in coal were massive and veinlets. It seems that the bacteria took priority to remove the massive pyrite. The sulfur relative contents analysis from XANES showed that the elemental sulfur (28.32%) and jarosite (18.99%) were accumulated in the biotreated residual coal. However, XRD and XANES spectra of residual pyrite indicated that the sulfur components were mainly composed of pyrite (49.34%) and elemental sulfur (50.72%) but no other sulfur contents were detected. Based on the present results, we speculated that the pyrite forms in coal might affect sulfur biooxidation process.

## 1. Introduction

Coal has the most abundant reserves and is always the most important energy on earth, which accounts for more than 24% in energy production in the world [1]. Nevertheless, direct combustion coal leads to the emission of sulfur oxides, which can cause adverse effects on environment [2]. Today, China is the largest emitter of SO_2_, and about 25.9 million tons of SO_2_ were emitted in 2006 [3], and thus precombustion desulfurization of coal is essential.

Sulfur is present in coal mainly in three forms: pyritic, organic, and sulfate; in addition, a small amount of sulfur may also be associated with coal in elemental form [4, 5]. Compared to the conventional physical and chemical desulfurization method, biodesulfurization can selectively oxidize organic sulfur and inorganic sulfur in coal and even remove the finely disseminated pyrite in coal matrix [6, 7]. Mesophilic, moderately thermophilic, and extremely thermophilic microorganisms exhibit the ability to enhance pyrite oxidation and its conversion to soluble, easily washed-out compounds [6, 8]. Among them, the mesophilic *Acidithiobacillus ferrooxidans *(*A. ferrooxidans*) is the most frequently applied bacterium to remove pyrite from coal [6, 9, 10]. 

Pyrite in coal can be found either as macroscopic occurrences or microscopic forms, and the detailed forms of which most commonly occurred as massive, disseminated, thin, and platy in cleats/fractures, cell fillings, pyrite veins, and so on [11, 12]. It must be mentioned that a present study showed that the pyrite formation could also influence the coal's desulfurization. Singh et al. [4] found that the bacterium *Pseudoxanthomonas* sp. had the lower ability to remove disseminated and framboidal pyrite than cavity filling forms. And the reason of that might be caused by their small size, complicated structure, and their highly scattered occurrences. Concerned about the mechanism of pyrite biooxidation, two possible ways, the indirect mechanism and the direct contact mechanism, have been proposed in the past three decades [13–15]; however, it has not yet fully been elucidated. Furthermore, some researches proposed a combination of direct and indirect mechanism was probably responsible for its oxidation [16, 17]. During its oxidation, the pyrite is degraded via the main intermediate thiosulfate and consequently oxidized via tetrathionate and other polythionates, finally to sulfate with elemental sulfur and jarosite being a side or precipitation product [14, 18, 19]. Many investigators agreed that the deposited jarosite and the sulfur-rich layers onto the surface of pyrite might prevent the contact of the microorganisms and hinder its oxidation process [20–22]. Based on the above description, the pyrite forms in coal are various; furthermore, the biooxidation of which remains disputant. 

The main objective of this work is to compare the biodesulfurization of coal and bioleaching coal's pyrite with *A. ferrooxidans*. In order to identify the mineralogical transformation during the process and how they affect the sulfur-removal efficiency, the morphology of mineral surface, mineral composition, and sulfur speciation on mineral surface was analyzed by combined techniques such as SEM, XRD, and sulfur K-edge X-ray absorption near edge spectroscopy (XANES), respectively. Chemical measurements such as pH, *Eh*, and concentration of iron in leaching solution were periodically monitored. Also, the proximate analysis, total sulfur, and sulfur contents of coal samples were identified according to the standard procedure.

## 2. Material and Method

### 2.1. Strain Culture, Coal, and Coal's Pyrite Samples

*A. ferrooxidans* was isolated by our laboratory, and it was cultured in basal medium supplemented with 44.5 g·L^−1^ ferrous sulfate. The pH of the medium was adjusted with 1 M sulfuric acid to 1.7. The *A. ferrooxidans* was cultured in 500 mL flasks containing 300 mL medium and incubated at 30°C with shaking at 170 rpm. The cultured solution was filtered through filter paper (pore size 10 *μ*m) to remove the jarosite sediments. Then cells in the filtrate were collected by centrifugation at 10,000 rpm for 20 min. The cell pellets were resuspended with the sterile basic medium and used in the next biodesulfurization and bioleaching experiment. The coal sample used in the experiment was collected from songzao mine in Chongqing, southwest of China. Prior to use, the coal was dried at ambient temperature, ground, sieved, and sterilized at 120°C for 20 min with the basal medium. The proximate analysis, total sulfur, and sulfur contents of coal samples were analyzed according to the national standards (GB/T212-2008, GB/T214-1996, and GB/T215-1996) of China, respectively, and their related data were shown in Table 1. The massive pyrite used in the experiments was picked up from the coal samples with naked eyes and the element component was analyzed with XRF (S 45.95%, Fe 42.48%, only the main components presented). The mineralogical composition of the coal and coal's pyrite samples were established by XRD (Bruker D8 Advance X-ray diffractometer, Cu K*α* 1 irradiation, *λ* = 1.5406 Å, 0.02 two-theta steps, and a count time of 2 seconds per step).

### 2.2. Biodesulfurization and Bioleaching Experiment

The biodesulfurization processes were carried out in 250 mL flasks with 100 mL basic salt medium, 10% W/V pulp density, particle size of −65 Tyler mesh, and the initial cell concentration was 1.0 × 10^6^ cells·mL^−1^, and processing time of 24 d. Every four days, the pH values, Eh (oxidation potential), and total iron concentration of biodesulfurization system were determined. At the end of the experiments, the residual coal samples were prepared with the method mentioned in the literature [23], and the proximate analysis, total sulfur, and the relative sulfur contents in the coal samples were then measured as the method mentioned in Section 2.1. For pyrite bioleaching experiments, 250 mL flasks containing 100 mL sterilized culture medium and 1.0 g pyrite (particle size of −65 Tyler mesh) were incubated with cells (the initial cells concentration 1.0 × 10^6^ cells/mL) on a rotary shaker at 170 rpm, 30°C. Leaching characteristics of the microorganisms were monitored by the determination the concentrations of total iron ions, pH,Eh, and the number of cells. Among them, the total iron ions were measured by atomic absorption spectroscopy. Triplicate leaches were carried out under identical conditions to ensure the reproducibility of the bioprocess experiments. Chemical treatment experiments with the culture medium were used as sterilized control.

### 2.3. Scan Electronic Microscopy Analysis

The original and residual coal and coal's pyrite samples were obtained and transferred to a 1.5 mL tube containing 1 mL glutaraldehyde (25%, V/V), and these samples were dehydrated and plated, and then introduced into SEM (JEOL JSM-6360 LV) chamber for scanning electronic observation. The coal and coal's pyrite samples processed with sterile medium were also analyzed.

### 2.4. Mineral and Sulfur Contents Analysis

For analysis of intermediate sulfur compounds, several experiments were stopped at different times and aliquots of 1 mL samples were transferred into 1.5 mL tube and then centrifuged at 10,000 rpm, 10 min. Finally, the samples were frozen with liquid N_2_, frozen dried, and stored in anaerobic box at −20°C until XRD and X-ray absorption near edge spectroscopy (XANES) test. XANES were recorded on 4B7A beamline at Beijing Synchrotron Radiation Facility. The spectra of the sulfur containing model compounds including pyrite, sulfate zinc, elemental sulfur, jarosite, and dibenzothiophene (DBT) were obtained as performed before [24]. The detailed measurements process and data calculation were performed as our previous report [24]. 

## 3. Results and Discussion

### 3.1. Coal Biodesulfurization and Coal's Pyrite Bioleaching Characteristics

**Figure 1:**
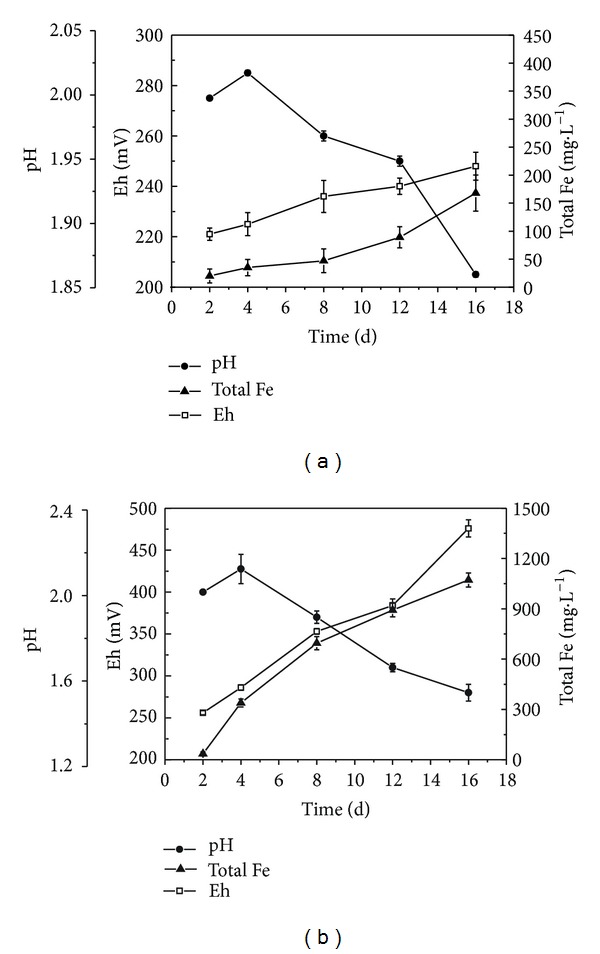
The biooxidation characteristics of coal without (a) and (b) with* A. ferrooxidans.*

Figure 1 shows the pH value, Eh, and total iron concentration changing behavior during the coal's desulfurization by sterile control (Figure 1(a)) and bacteria (Figure 1(b)). Compared with the sterile control, *A. ferrooxidans* significantly promoted pyritic sulfur oxidation, and the concentrations of total iron were 0.17 g·L^−1^ and 1.07 g·L^−1^, respectively. The proximate analysis and sulfur forms in coal samples before and after processing with cells are presented in Table 1. The total sulfur concentration in the raw and residual coal changed from 2.49% to 1.23%, and the elimination rate was 50.6%. Among them, the removal of pyrite was up to 69.9% after 16 days of processing. The bacteria had no ability to remove the organic sulfur in coals. The removal rate of pyrite sulfur was in agreement with the total iron ions concentration of culture solution. Both results showed that the coal's biodesulfurization was mainly caused by pyrite biooxidation. The Eh of culture solution increased with the total iron concentration, which indicated that the high Eh was helpful for iron oxidation.

**Figure 2:**
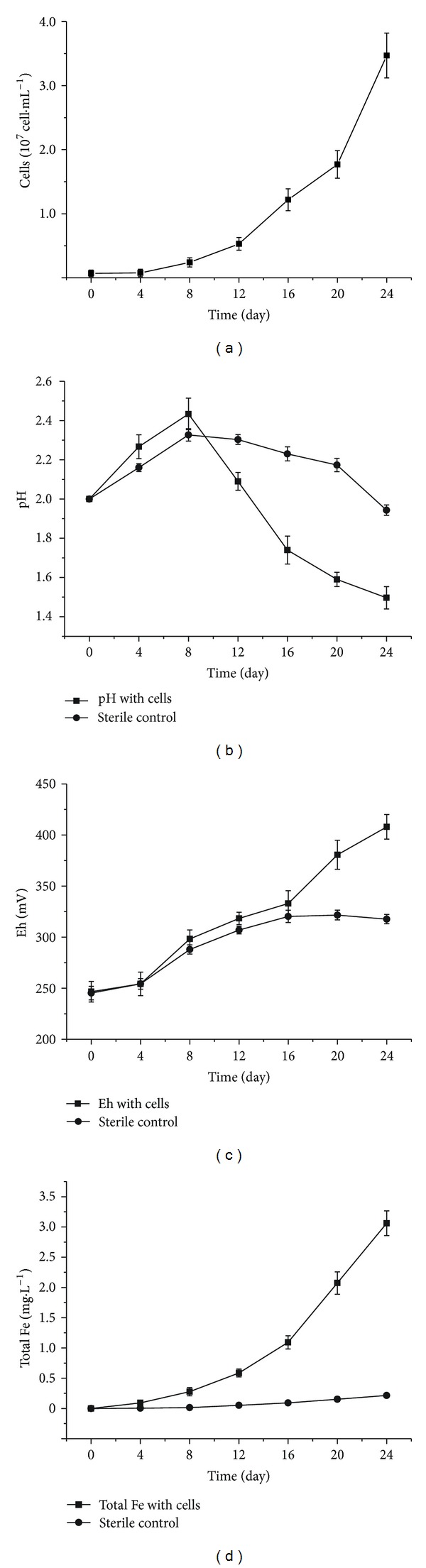
Leaching characteristics of coal's pyrite with *A. ferrooxidans *cells and sterile control (■: cells, *⚫*: sterile control).

The bioleaching characteristics of coal's pyrite by *A. ferrooxidans* and sterile medium are shown in Figure 2. The cells number in the bioprocess system increased with time and reached a value of 3.6 × 10^7^cell·mL^−1^ on the 24th day (Figure 2(a)). During the process, the pH started to increase gradually then decrease slowly; however, it had differences in both solution systems (Figure 2(b)). The pH in the bioprocess system increased with time and reached a maximum on the 8th day (pH = 2.43) and thereafter decreased to a value of 1.49, while in the sterile controls the solution almost restored to the original pH. It presented the same phenomenon as the above coal's biodesulfurization process. It has been reported that the initial increase in pH at coal's biodesulfurization might be caused by carbonates dissolution [6]. However, in the pyrite bioleaching system the increase of pH was mainly caused by the consumption of protons. From the current results, we speculated that the oxidation of pyrite might consume more acid at the initial stage of process than coal's pyritic sulfur oxidation. The Eh of bioleaching solution increased along with the accumulation of Fe^3+^ both in the cells and sterile systems, with a final value of 408 mV and 317 mV, respectively (Figure 2(c)), which was lower than in earlier biodesulfurization process. It shows that the *A. ferrooxidans* has significantly promoted to the bioleaching pyrite, with a final total iron concentration of 3.06 g·L^−1^. However, there was only 0.21 g·L^−1^ of iron ions in the sterile control solution after 24 days of processing (Figure 2(d)). 

### 3.2. Coal and Coal's Pyrite Surface Morphology Analysis

**Figure 3:**
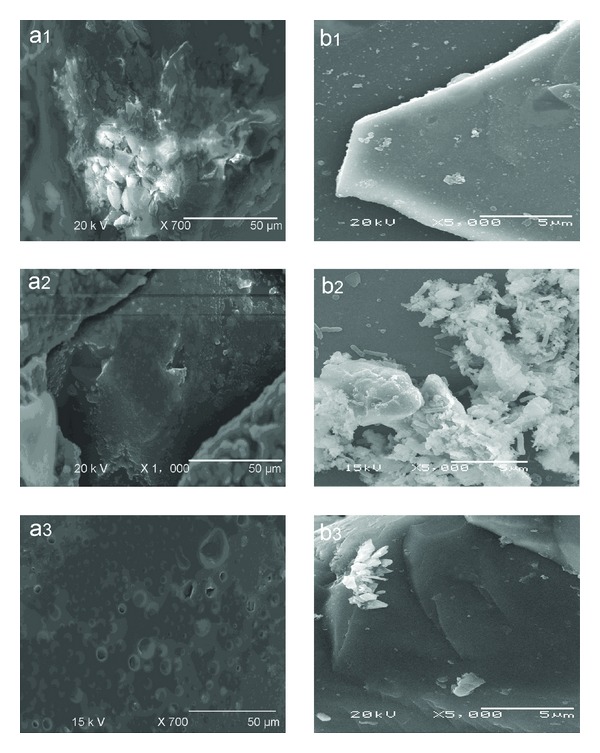
SEM micrographs of biodesulfurization of coal (a1) after 0 day, (a2) after 6 days and (a3) sterile control; bioleaching coal's pyrite (b1) after 0 day, (b2) after 24 days, and (b3) sterile control after 24 days.

The SEM graphs of coal and coal's pyrite in the bioprocess system are shown in Figure 3((a1)–(b3)). The major forms of pyrite in coal samples were massive and veinlets (Figure 3(a1)). After 16 days of bioprocessing, the massive pyrite in the coal samples decreased evidently (Figure 3(a2)). However, the coal of sterile control almost remains unchanged (Figure 3(a3)). According to Singh et al.'s [4] report, the bacterium presents strong ability to remove massive pyrite than microscopic forms. It seems that the same phenomenon occurs in our present work. As shown in Figure 3(b1), the pyrite surface morphology obviously changes during the bioprocess experiment. The SEM micrograph showed that the pyrite surface was smooth before processing, but after 12 days of bioprocessing, visible corrosion of pyrite was observed, and numerous bacteria were adsorbed on concave parts of the mineral surface (Figure 3(b2)). In contrast, the pyrite surface was a little changed after treated for 24 days by the sterile control (Figure 3(b3)).

### 3.3. Sulfur Speciation Transformation of Coal Biodesulfurization and Coal's Pyrite Bioleaching

**Figure 4:**
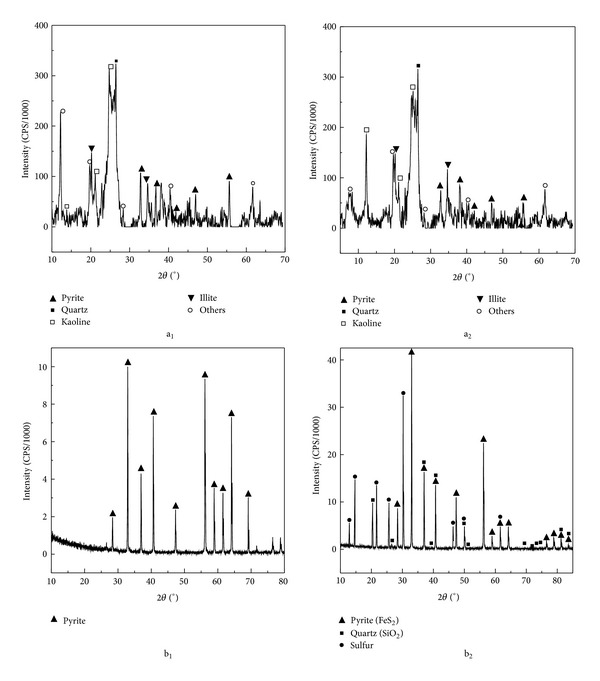
XRD analysis of coal before (a_1_) and after (a_2_); pyrite before (b_1_) and after (b_2_) processed with *A. ferrooxidans.*

 X-ray diffraction spectra of raw and residual coal samples are shown in Figures 4(a_1_) and 4(a_2_). Mineral contents of coal samples are mainly composed of pyrite, quartz, kaolin, and illite. After processing with bacteria, the pyrite and kaolin peaks reduced subtly. It seems that the decrease of ash of coal samples was mainly caused by the reduction of pyrite. In the XRD analysis of the original pyrite, residuals in bioprocess experiments by *A. ferrooxidans *are shown in Figures 4(b_1_) and 4(b_2_). It indicated that there were characteristic diffraction peaks of sulfur and silicon dioxide in residuals of the bioprocess system in addition to pyrite (Figure 4(b_1_)). In addition, according to the SEM analysis in Figure 3(b2), we speculated the peripheral mineral in the leaching products might be elemental suflur. Shi and Fang [25] found a porous layer of elemental sulfur formed on the surface when using SEM and X-ray energy dispersion spectroscopy to research the leaching of chalcopyrite by *A. ferrooxidans*.

**Figure 5:**
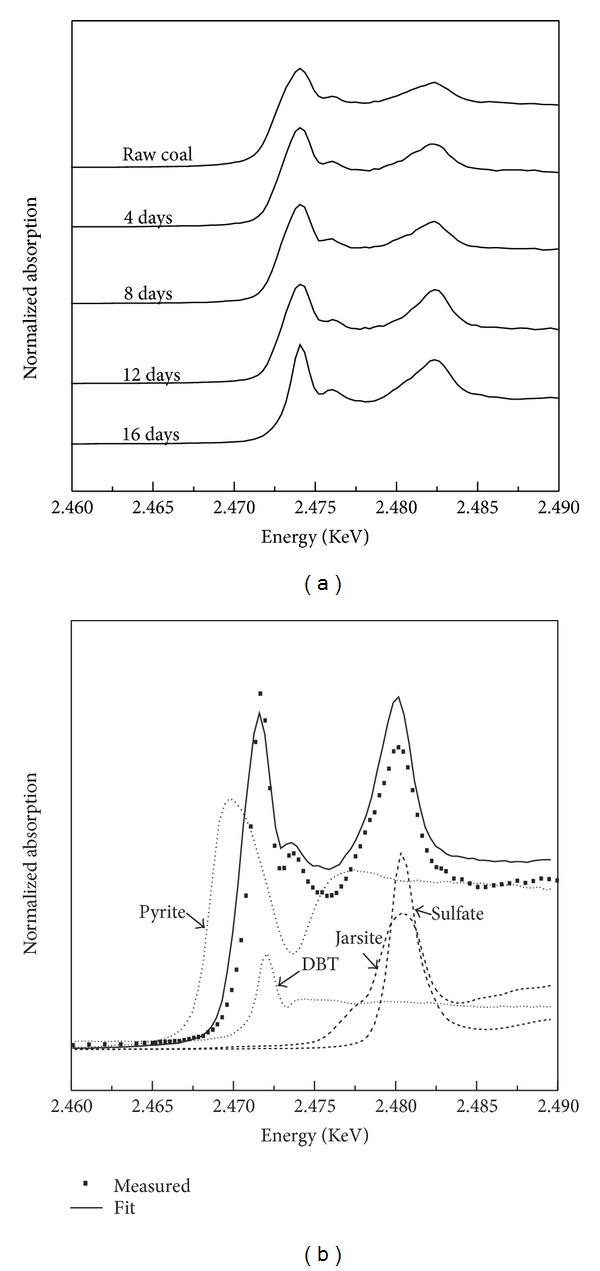
Normalized sulfur K-edge XANES spectra of coal samples processed with *A. ferrooxidans.*

Figure 5 and Table 2 show the sulfur speciation transformation during the coal's biodesulfurization process and the relative sulfur components of raw and residual coal. It shows that there are significant changes at the absorption peaks' width and intensity after being bioprocessed with cells (Figure 5(a)). The absorption peak at 2.4804 KeV assigned to sulfate absorption changed widely and strongly as the bioprocessed time. The fitted XANES spectra from the residual coal biotreated 16 days (Figure 5(b), Table 2) revealed that these sulfur contents were essentially changed. Compared with the raw coal, the pyrite, sulfate, and DBT in residual coal were 35.67%, 28.32%, and DBT 13.86%, respectively. Furthermore, there appears jarosite (18.99%) in the residual coal. Nevertheless, the organic sulfur contents of DBT almost remains constant, only changed from 14.34% to 13.86%. Based on the current results, the bioprocessed coals increased the oxidized sulfur contents and the pyrite was preferentially removed by the cells.

**Figure 6:**
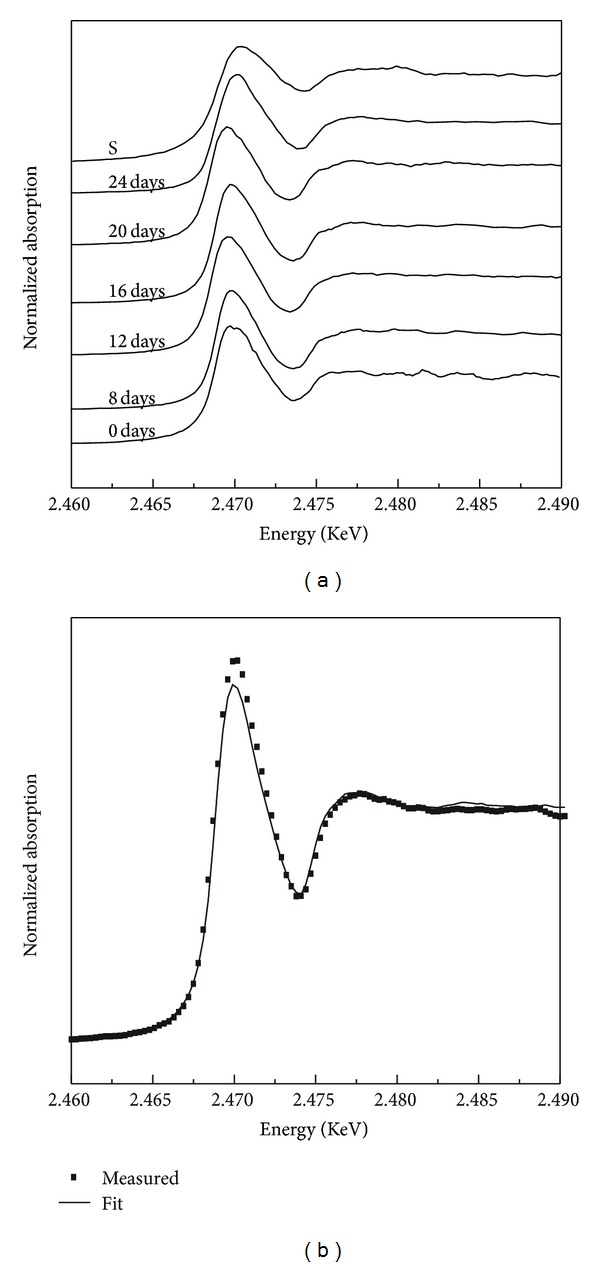
Normalized sulfur K-edge XANES spectra of pyrite leaching with *A. ferrooxidans.*

A lot of investigations have focused on the solution mechanism of pyrite; however, the detailed chemical speciation on the mineral surface is always open to dispute. The sulfur K-edge XANES spectra of pyrite during leaching process by *A. ferrooxidans* are shown in Figure 6(a). The sulfur K-edge XANES spectra of pyrite bioleached in early days show almost similar absorption features to reference pyrite, in both edge position and intensity. However, as time goes on, the absorption edge gradually shifts to high-energy side and after 24 days of processing there appears a significant peak at 2.4702 keV. In contrast, the sulfur K-edge XANES spectra of pyrite processed with the sterile controls did not show change in the absorption features after leaching for 20 days (data not shown). Combined with the XRD spectra and the sulfur K-edge XANES of sulfur, it can be concluded that the absorption peak at 2.4702 keV is mainly derived from the sulfur accumulated on the surface of bioprocess pyrite. After being fitted with the standard compounds, the spectra show some new information.  Figure 6(b) shows the fitted curve of the bioprocess residual. The results suggest that there were 49.3% pyrite and 50.7% sulfur in the sample processed after 24 days by *A. ferrooxidans* but there was no other sulfur speciation identified. Our results were in agreement with the reports from the electrochemical oxidation behavior of pyrite bioleaching by *A. ferrooxidans *[26], in which the oxidation reaction of pyrite was divided into two steps: the first reaction step involves the oxidation of pyrite to S, and the second reaction step is the oxidation of S to SO_4_^2−^, and the passivation layer on the surface was mainly sulfur. However, Liu et al. [27] found that there was amount of jarosite but no elemental sulfur was detected in the pyrite leaching residues by *A. ferrooxidans*. So they supported that there was a thiosulfate mechanism in *A. ferrooxidans* bioleaching pyrite, where the thiosulfate led to sulfate without elemental sulfur formation. Colling et al. used *A. ferrooxidans* to bioleaching the pyrite present in coal tailings to produce a ferric sulfate coagulant. It seems that the main product was sulfate but no other sulfur contents were reported [28]. The current XANES results revealed that the relative concentration of pyrite sulfur decreased and elemental sulfur occurred in both biodesulfurization and bioleaching system. Furthermore, in the former residual coal samples, the jarosite was identified but not found in the latter process. In our experiment, the biotreated pyrite samples were washed three times with ionic water to remove the sulfate in the culture medium before the spectrum analysis. We speculated that the sulfate speciation on the mineral surface might be cleaned off by the washing treatment. On the other hand, in the present work, the massive pyrite samples were used in bioleaching experiments, while both massive and microscopic pyrites were processed during the coal's biodesulfurization. Because of the different affinity of pyrite forms with coal's liptinite and inertinite macerals, the bacteria presented distinct biodesulfurization rate [4]. Based on our present work, it seems that the pyrite forms might affect the mineralogical composition and sulfur contents during the coal's biodesulfurization and coal's pyrite bioleaching.

## 4. Conclusion

 To know the biodesulfurization of coal and the bioleaching of coal's pyrite by *A. ferrooxidans*, SEM, XRD, and sulfur K-edge XANES spectroscopy were involved to analyze the mineral composition and sulfur speciation evolution process. The result showed that the bacteria significantly promoted the biodesulfurization of coal and bioleaching of coal's pyrite. *A. ferrooxidans* preferentially removed the massive pyrite but has almost no oxidation ability on organic sulfur. The data from sulfur XANES spectra of biodesulfurization coal revealed that the jarosite and elemental sulfur were accumulated. On the contrary, only elemental sulfur was present, but there was no other sulfur speciation identified on the coal's pyrite surface. We suspected that the different pyrite forms might also influence its biooxidation process.

## Figures and Tables

**Table 1:** Characteristics of the coal samples (wt%).

Sample	Proximate analysis (dry basis)			Sulfur components			
	*M*	Ash	*V*_*m*_	Total	Pyritic	Sulfate	Organic
Raw coal	7.88	21.00	8.64	2.49	1.46	0.66	0.37
Residual coal	6.42	8.27	7.86	1.23	0.44	0.45	0.34

*M*  stands for moisture. *V*_*m*_ stands for volatile matter.

**Table 2:** The fitting results of S K-edge XANES spectra of measure sample with different reference spectra.

Sample	Percentage of contribution of standard spectra (%)				
	Pyrite	Elemental sulfur	Sulfate	Jarosite	DBT
Raw coal	45.64	0	23.57	0	14.34
Residual coal (16 days)	35.67	28.32	10.16	18.99	13.86
Raw pyrite	100	0	0	0	0
Residual pyrite (24 days)	49.34	50.72	0	0	0
